# A novel algorithm to determine ventilation parameters during cardiopulmonary resuscitation using pneumotachography waveform data

**DOI:** 10.1016/j.resplu.2026.101238

**Published:** 2026-01-21

**Authors:** Johan Mälberg, Jeroen A. van Eijk, Lotte C. Doeleman, Patrick Schober, Hans van Schuppen, David Smekal, Sten Rubertsson, Douglas Spangler

**Affiliations:** a Department of Surgical Sciences‑Anesthesia and Intensive Care, Uppsala University, Uppsala, Sweden; b Amsterdam UMC location Vrije Universiteit Amsterdam, Anesthesiology, Boelelaan 1117, Amsterdam, the Netherlands; c Amsterdam UMC location University of Amsterdam, Anesthesiology, Meibergdreef 9, Amsterdam, the Netherlands; d Amsterdam Public Health, Amsterdam, the Netherlands

**Keywords:** Cardiopulmonary resuscitation, Ventilation, Chest compressions, Tidal volume, Reversed airflow, Signal artefacts

## Abstract

**Background:**

A major barrier to the analysis of ventilation waveform data collected during CPR is the presence of artefacts caused by chest compressions. This study describes the development and evaluation of an algorithm to extract parameters regarding ventilation volume, pressure, and frequency from pneumotachography waveform data collected during ongoing simulated CPR.

**Method:**

Ventilation waveform data was collected from a pneumotachograph connected to the respiratory circuit of a ventilator and a test lung. Both regular ventilation and ventilation during simulated CPR were used to develop the algorithm. A grid search was employed to optimize the algorithm parameters compared to the ventilator settings. The parameters were then manually tuned using clinical data from ventilation during CPR. The performance of the algorithm was described in terms of the median error vs. the known ventilator settings in the simulated data.

**Results:**

Compared to the ventilator settings, the largest systematic errors of the algorithm was an overestimation of peak pressures during asynchronous CPR (median error of 3 (IQR 0.3–5.8) cmH_2_O), and an underestimation of inspiratory volumes during synchronous CPR (median error 46 (IQR −76 to 10) ml).

**Conclusion:**

In an experimental setting, the developed algorithm provides a novel solution to measure ventilation parameters during ongoing chest compressions. The algorithm is freely available under an open-source licence for use and further development. Further studies will be needed to validate the algorithm.

## Introduction

Ventilation during cardiopulmonary resuscitation (CPR) remains insufficiently studied, and although studies show that optimizing ventilation can potentially improve outcomes,^1^, ^2^, ^3^, ^4^ current advanced life support (ALS) recommendations still rely largely on expert opinion.^5^, ^6^, ^7^, ^8^ A major challenge in developing evidence-based recommendations has been the difficulty of systematically measuring ventilation during CPR, especially since manual ventilation is commonly used.

While capnography is a well-established method for measuring end-tidal carbon dioxide (etCO_2_) during CPR^9^ and estimating ventilation rates, pneumotachography has traditionally been used to measure ventilation in non-CPR settings like anesthesia and intensive care.^10^, ^11^ This technique can provide detailed information about flow, pressure, and volumes, many of which are directly relevant during resuscitation.

Recent advances in monitoring devices have demonstrated that measuring manual ventilation is feasible in the prehospital setting.^12^, ^13^, ^14^ However, during CPR, ventilation parameters are highly dynamic due to inspiratory and expiratory flow in the airways caused by chest compressions and decompressions. Various artefacts can complicate signal processing, making it challenging to obtain accurate data.^15^, ^16^ This issue is particularly pronounced during asynchronous ventilation, where ventilations are provided during ongoing chest compressions, which can lead to both reversed airflow^17^ and sudden increases in airway pressure.^18^

Therefore, when analyzing pneumotachography in the CPR setting, specific adaptations are required to ensure accuracy. The aim of this study was to develop an algorithm capable of extracting accurate ventilation parameters during active ventilation from signals recorded during CPR using a pneumotachography device.

## Methods

This was an experimental algorithm development study with the purpose to extract ventilation parameters from pneumotachography waveform data during simulated CPR. The collection of patient data used in this study was approved by the Swedish Ethical Review Authority (Dnr 2018/531) and the Medical Ethics Review Committee of Amsterdam UMC (approval number 2023.0998) and conducted in accordance with the Declaration of Helsinki.^19^

### Materials

Data was collected using a portable pneumotachograph (Fluxmed GrH MBmed, Buenos Aires, Argentina) coupled with a capnograph (Capnostat 5, Philips, Amsterdam, the Netherlands), which were both connected to a portable computer storing the data. This setup will subsequently be referred to as “the device”. The device samples flow, pressure, and CO_2_ at 256 Hz and outputs a waveform data file for analysis. With the device came proprietary software for extraction of ventilation parameters from waveform data, from here on out referred to as the “standard method”.

### Data collection

Two different datasets of waveform data from ventilations were used in the development and evaluation of the algorithm. The main dataset used in this study consisted of ventilator data, both with and without simulated CPR. The other dataset consisted of clinical data from the forthcoming Cardiac Arrest ventilation (CAvent) study.^20^

**Fig. 1:**
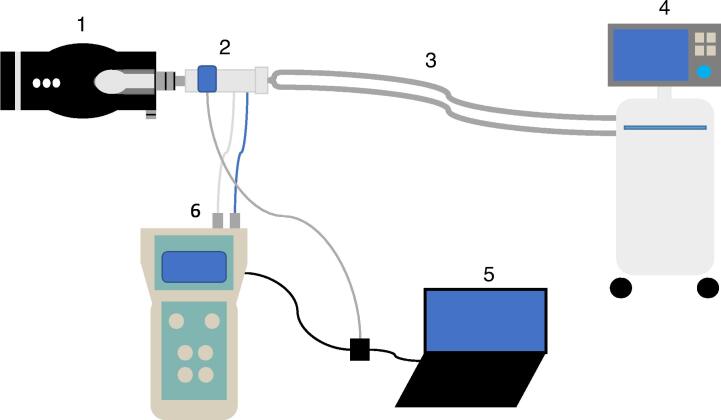
**Schematic illustration of the setup (not to scale). 1: Test lung, 2: Flow sensor and capnograph, 3: Respiratory circuit, 4: Ventilator, 5: Portable computer, 6: Pneumotachograph device**.

In the main dataset, used for development and evaluation of the algorithm, the device was connected to the ventilation circuit between a test lung (Smartlung Adult, IMT. Analytics, Buchs, Switzerland) and a ventilator (of which two different models were used, Maquet Servo-i or Maquet Servo-u, Getinge, Gothenburg, Sweden). Fig. 1 shows a schematic illustration of the experimental setup.

Different settings were used on the ventilator to enable evaluation of a wide variety of different volumes (177–612 ml), pressures (13–45 cmH_2_O) and ventilation rates (10–30 per minute) in both volume-controlled and pressure-controlled mode. Supplemental file 1 describes all tests and their corresponding settings and modes. Other settings were left unchanged including maximum airway pressure (pMax, 60 cmH_2_O), trigger (disabled) and positive end-expiratory pressure (PEEP, 0 cmH_2_O). Both ventilations without CPR and during simulated CPR were used. To simulate CPR, the test lung was manually compressed (by the same person) at a metronome aided rate of 110 compressions/minute with a depth that generated oscillations in flow and pressure waveforms visually similar to those observed in human CPR. Ventilations were either provided asynchronous to the chest compressions or synchronous. During synchronous mode, two insufflations were provided by a manually started ventilator in the pause after 30 compressions. Directly after the second exsufflation, compressions were resumed, and ventilations were paused by using an expiratory breath-hold maneuver on the ventilator. Airway leakage was also simulated by adjusting the built-in leakage valve on the test lung.

The CAvent dataset was used for manual fine tuning of threshold parameters. The CAvent study was an observational study using the same device as employed in this study. In the CAvent study, the device was connected between the airway management device (either a mask, a supraglottic airway device or an endotracheal tube) and the self-inflating bag. The cohort included Swedish and Dutch cardiac arrest patients receiving CPR by advanced life support (ALS) providers using manual ventilation. The study included 28,120 ventilations in 241 patients, of which 69.3% were male and the mean age was 68 (interquartile range (IQR) 56.0–77.0). The mean height and length of the included patients were 1.75 m (IQR 1.70–1.80 m) and 80.0 kg (IQR 70.0–90.0 kg), respectively.

### Algorithm development

**Fig. 2:**
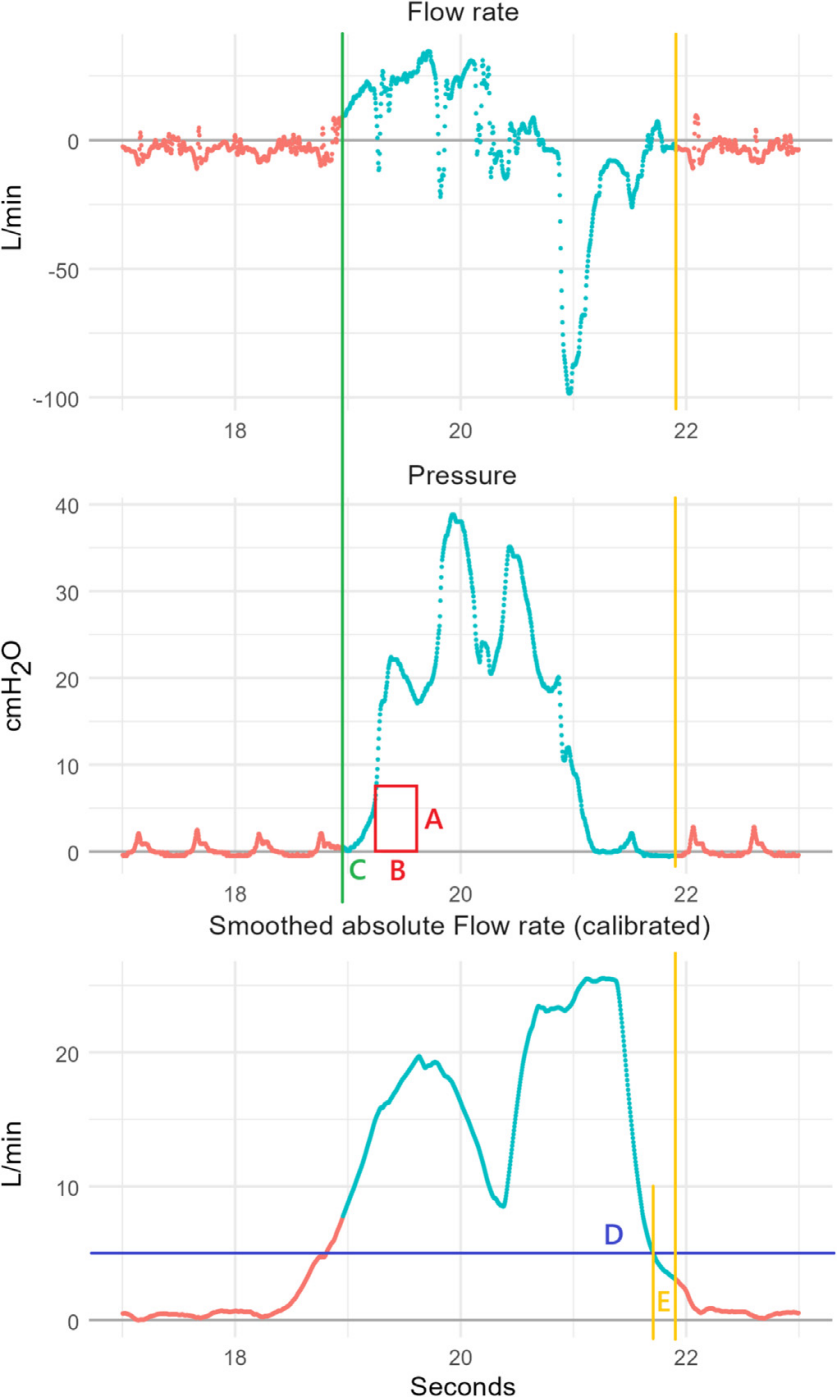
**Example flow and pressure curves during asynchronous CPR, including representations of the parameters evaluated in developing the ventilation identification algorithm. The turquoise part of the waveforms indicates the active phase and the red parts the inactive phase. The yellow line represents the point at which the inactive phase begins. (A) Pressure threshold, (B) Pressure duration, (C) Start of insufflation, (D) Inactive phase absolute flow rate threshold, and (E) Inactive phase delay**. (For interpretation of the references to color in this figure legend, the reader is referred to the web version of this article.)

Raw waveform data regarding inspiratory pressure and flow rates from the device was imported into R v. 4.4.2. (R Foundation for Statistical Computing, Vienna, Austria). An algorithm was developed to detect the start of each insufflation by evaluating the sustained positive pressure over a given period (Fig. 2, rectangle with dimensions AB). Optimal dimensions must be selected to be sufficiently large to exclude compression artefacts, but as small as possible to detect true insufflations with high sensitivity. An additional parameter to define the insufflation as beginning slightly prior to the start of the rectangle was included to capture airflow occurring at the start of the insufflation (Fig. 2, line C).

A period representing the active phase of ventilation was defined from the start of insufflation to the end of detectable exsufflation. The end of this period was defined as the first time point at which both, (1) the absolute flow rate, averaged over one second, was reduced below a given threshold, and (2), the ventilation pressure was below the pressure threshold A (Fig. 2, line D). Because ongoing CPR causes absolute flow rates to be continually elevated, the absolute flow rate was calibrated by subtracting the minimum flow rate value, measured in the first 6 s after the start of the active ventilation (to prevent periods of CPR cessation in synchronous compression regimes from being used as the baseline). To ensure complete capture of expiratory volume, the end of the active ventilation phase was extended by a fixed delay beyond point D (Fig. 2, line E). Inspiratory volume was calculated as the summation of the positive flow over time during the active ventilation phase, and expiratory volume as the summation of the negative flow over time during the same phase.

### Parameter selection

To select these parameters, a grid search was performed across a range of potential values to identify a combination which minimized the average standardized absolute deviation of volume (inspiratory and expiratory), peak pressure, and ventilation rate (for continuous ventilations) as compared to the ventilator settings. To weight each measure equally, the absolute deviation from the ventilator settings for each measure was standardized based on the standard deviation of the measure across all test runs. In addition to these non-tuned parameters, some rule-based parameters were included based on domain knowledge: a minimum ventilation duration of 0.3 s was selected to prevent CPR artefacts from resulting in counting multiple ventilations within a single true ventilation. While the measurements of CO_2_ could not be validated using the test lung, code to capture CO_2_ is included, which is measured as the maximum CO_2_ value at any time during the ventilation (active or inactive), including a delay parameter to ensure that the final expired CO_2_ is not attributed to the subsequent ventilation. CO_2_ outputs are provided in the open-source code, but were not used in parameter tuning, or for the primary analyses in this study.

The grid-search identified optimal parameters in the simulated CPR data were then applied to actual patient data from the CAvent study for qualitative assessment. The clinical data was reviewed for face validity by two of the authors (JM and DSp). After reviewing 20 randomly selected patients, both reviewers agreed that the thresholds selected in the grid search were too low, based on the apparent size of artefacts in the clinical data. Therefore, higher pressure and absolute flow thresholds than identified as optimal in the grid search were selected to be used in the evaluation of the algorithm, with the remaining parameters selected based on the grid search. The algorithm parameters, the values evaluated in the grid search, theoretically optimal values, and manually tuned values following application on real-world data are presented in Table 1. Supplemental file 2 provides examples of waveforms from the clinical data and the results of the manual tuning of the threshold parameters (under “Additional accuracy measures”).

**Table 1:** Parameters evaluated in grid search.

	**Parameter name**	**Grid search parameter values**	**Optimized parameter values**	**Manually adjusted parameter values**
A	Pressure threshold	1, 2, 5, 8, 10 cmH_2_O	2	8*
B	Pressure duration	0.1, 0.2, 0.3, 0.4, 0.5 s	0.3	0.3
C	Ventilation lead	0, 0.1, 0.2, 0.3, 0.4, 0.5 s	0.1	0.3
D	Absolute flow threshold	−1 (no inactive phase), 1, 5, 10 L per minute	1	5*
E	Inactive delay	0, 0.1, 0.2, 0.3 s	0.2	0.2

* Values held constant upon assessment of algorithm in clinical data set.

### Statistical analysis

Patient data are presented as percentages for categorical variables and medians with IQR (Q1-Q3) for continuous variables. The final algorithm was evaluated at the individual ventilation level in terms of the difference between the ventilator-set or measured value (depending on ventilator settings), and the algorithm-based measures of inspiratory and expiratory volumes, peak pressure, and ventilation rate. As the clinical data did not have known values to compare with, only the simulated ventilation data could be used in this evaluation. The performance of the algorithm was described using median values and IQR, and percentage error per the Bland-Altman method.^21^ Data were visualized using histograms and example raw waveform data. Additional quantifications of the performance for both the algorithm presented here and the standard method, including Lin’s Concordance Correlation Coefficient,^22^ Root Mean Square Error, proportion of ventilations with <10% error, and Bland-Altman plots are available in Supplemental file 2. All algorithm development and analyses were performed using R v 4.4.2. All code necessary to reproduce and replicate the results are available in Supplemental file 2, and the simulated ventilation data is available in an online repository.^23^

## Results

**Fig. 3:**
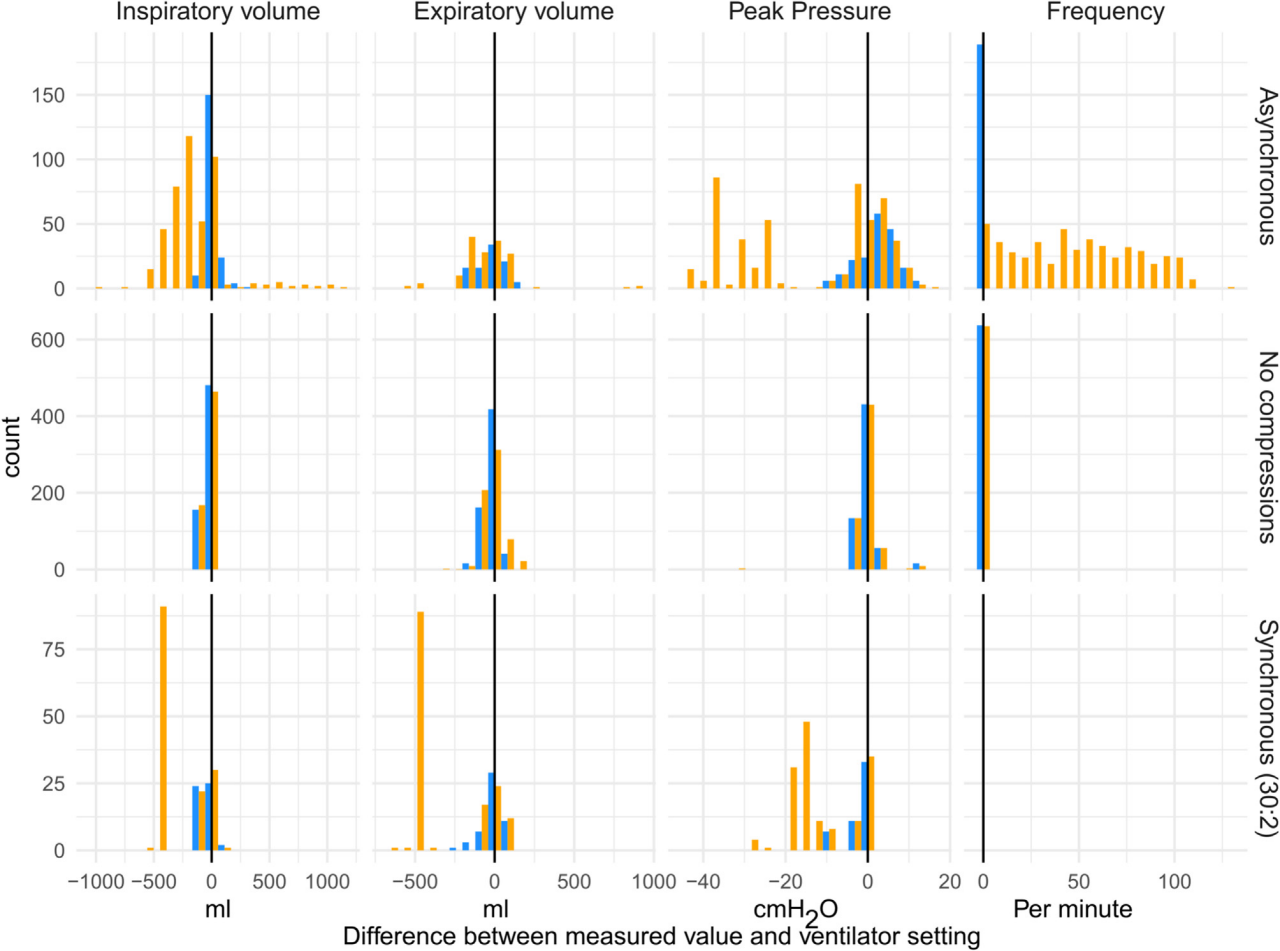
**Histogram of errors between measured parameters and ventilator setting. The black lines indicate the ventilator setting. Yellow bars represent the standard method used by the device, while blue bars represent the measures estimated by the algorithm. Count refers to the number of ventilations in each histogram bin**. (For interpretation of the references to color in this figure legend, the reader is referred to the web version of this article.)

Simulated ventilations were performed in 37 runs of two minutes each, yielding a total of 876 ventilations involving various combinations of ventilator settings with and without ongoing simulated CPR. Table 2 reports differences in median ventilator settings and median measured values by the algorithm across all runs. Deviations from the ventilator settings were an overestimation of pressure in asynchronous ventilation by 3 (0.3–5.8) cmH_2_O, corresponding to a 10.3% error, and an underestimation of inspiratory volumes in synchronous ventilation by −46 (−76 to 10) ml, corresponding to a 13.5% error. The identified ventilation frequency matched the ventilator setting with a deviation of 0.1%. Comparisons between the standard model and the ventilator settings can be found in Supplemental file 2. Fig. 3 shows differences visually, including both those from the algorithm and the standard method.

**Table 2:** Descriptions of ventilator-set and algorithm measured ventilation parameter values.

**Ventilation type**	***N* runs**	*N* breaths*	**Measure**	**Set value median**	**Measured value median**	**Difference median (IQR)**	Difference %**
Asynchronous	10	189	Inspiratory volume (ml)	400	396	5 (−28 to 32)	1.1
		92	Expiratory volume (ml)	413	399	−8 (−43 to 68)	−1.6
		189	Peak pressure (cmH_2_O)	36	37.1	3 (0.3–5.8)	10.3
		189	Frequency (per minute)	10	10.0	0 (0–0.1)	0.1

No compressions	20	637	Inspiratory volume (ml)	500	445	−10 (−55 to 22)	−3.3
		637	Expiratory volume (ml)	399	388	−22 (−44 to 24)	−5.8
		637	Peak pressure (cmH_2_O)	23	22	0.1 (−1.4 to 0.5)	0.5
		637	Frequency (per minute)	20	20.0	0 (0–0.1)	0.1

Synchronous (30:2)	7	51	Inspiratory volume (ml)	500	423	−46 (−76 to 10)	−13.5
		51	Expiratory volume (ml)	299	348	−5 (−30 to 28)	−0.8
		51	Peak pressure (cmH_2_O)	22	21.4	−1 (−1.9 to −0.2)	−4.9
		0	Frequency (per minute)	***	***	***	***

* Some runs were excluded from evaluation of expiratory volumes due to measurement errors by the ventilator.

** The percentage difference is calculated as the difference between the set and measured values, divided by their average value.

*** Synchronous ventilations were initiated manually, and no ventilation setting to compare with is available*.*

**Fig. 4:**
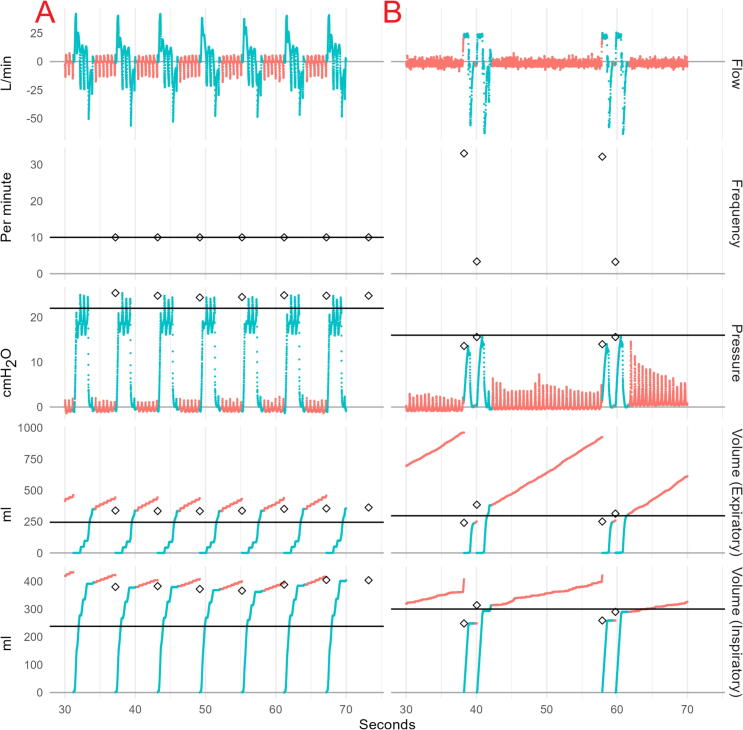
**Illustration of raw waveforms and ventilator parameters. Panel A shows asynchronous ventilations and panel B show synchronous ventilations. Turquoise portions of the waveforms were identified by the algorithm as active periods of ventilations, while red periods are considered inactive. Ventilator settings are denoted by black horizontal lines, while algorithm measured values are denoted by black diamonds at the start of each ventilation**. (For interpretation of the references to color in this figure legend, the reader is referred to the web version of this article.)

Fig. 4 illustrates waveforms for two pertinent examples where measurement errors arise in our algorithm. In most cases, the algorithm corresponds with the start and end of the active phase of each ventilation, and measured values correspond visually with the waveforms. As seen in panel A, ongoing CPR during ventilation leads to a sawtooth pattern resulting in greater volumes and higher peak pressures measured by the algorithm than by the ventilator. In panel B, a relatively slow increase in pressure leads to the ventilation being identified late and clipping the start of the ventilation, leading to an underestimation of inspiratory volumes, particularly for the first of the two ventilations.

## Discussion

In this study, we present a novel algorithm capable of extracting ventilation parameters from data simulating ventilation during ongoing CPR in an experimental setting. The algorithm, in the tested setting, matched ventilation parameters with an average difference of at most 13.5% (underestimating inspiratory volume during synchronous compressions by 56 ml) and 10.3% (overestimating peak pressures during asynchronous compressions by 3 cmH_2_O). While these discrepancies should be considered when evaluating the results of research based on this algorithm, it constitutes the first reasonably accurate and transparent method for estimating ventilation parameters from raw waveform data during ongoing CPR. The ventilation parameters estimated by the algorithm also outperformed the standard method in both synchronous and asynchronous CPR, and offer a slight improvement in performance in the setting of ventilation without CPR.

There are numerous challenges when measuring ventilation during CPR that necessitated the development of this algorithm. The reversed airflow and oscillating airway pressures caused by the chest compressions^15^ cause significant impact on standard measurements of volume, pressure and ventilation frequency if not accounted for. The approach to minimizing this impact in our algorithm was to use positive airway pressure instead of flow to detect ventilations, and implementing a threshold value to trigger detection. The threshold had to be high enough to exclude pressure changes from the chest compressions but low enough to not miss true positive pressure ventilations. This also enabled the algorithm to filter out negative airway pressure due to spontaneous breathing. Although detection of spontaneous breathing could be clinically important, the aim for this algorithm was to accurately measure positive pressure ventilation. However, if spontaneous breathing during CPR is of interest, the parameters of the algorithm can be adjusted to detect breaths with negative airway pressures.

Reversed airflow also affects expiration measurements. If all the airflow during the ventilation pauses is included in expiration measurements, as proposed in a previous study,^15^ we found that it could produce erroneous minute volumes that are significantly elevated. Current literature suggests that tidal volume caused by this passive ventilation may be insufficient to overcome anatomical dead space, raising uncertainty about its meaningful contribution to gas exchange.^2^ Therefore, it could be argued not to include these fluctuations in flow when measuring active ventilation, which was the aim of our algorithm, and instead focus on the cessation of flow to identify when the expiration directly following an insufflation stops. However, it can be challenging to determine when this occurs due to the constant bi-directional flow. The presented algorithm distinguishes between an active phase of ventilation, and an inactive phase, when airflow and pressure is minimal, by employing a threshold for the smoothed absolute average of flow to determine when the expiration directly following an active insufflation ended. This enables an accurate estimate of the expiratory volume and flow while minimizing errors due to CPR artefacts, leakage during the passive ventilation, and any potential miscalibrations of the devices, while also facilitating an approximation of airway leakage during ventilation.

For the development of this algorithm, ventilator measurements were considered the gold standard against which the algorithm was compared. However, given that measurements occurred at different points in the flow, the differences could be due to leakage between the measurement devices at some point, or due to measurement errors in the ventilator, the occurrence of which has previously been shown during experimental CPR.^24^ It is not entirely clear whether pressure or volume controlled ventilations are most appropriate for simulating active (manual) ventilations, and data from both modes of operation were included in the data used for evaluation of the algorithm. Qualitatively, it appeared that in cases where the measures and true ventilator settings differed (such as the inspiratory volumes in panel A of Fig. 3), the algorithm captured the measured values suggested by the raw data adequately. Thus, some portion of the remaining error may stem from true discrepancies between how and where the device and ventilator measure flow and pressure.

The algorithm developed in this study approached ventilation parameters measured by a ventilator during simulated CPR with relatively small errors, which are not clinically relevant for adult patients. We suggest that the algorithm could be useful for research and, with further development and validation, can be included in ventilation review software for clinical purposes. The algorithm, as well as all data used in this evaluation, is available in an open repository to encourage further use and development, and parameters are easily adjusted if necessary.^23^

## Limitations

This study has some limitations. It is possible that the algorithm will lose performance when applied to new data from a different setup, outside the context of this single experimental setup. Additionally, as standard ventilator algorithms are not designed for measurements during CPR, this could affect the values used as the gold standard to compare with. Further evaluation with additional ventilation devices could be beneficial. Overfitting might have occurred due to the lack of a held-out test dataset. However, given the small number of free parameters in the algorithm, the degree of overfitting stemming from random variability in the data is likely to be small. Both issues are best addressed through the external validation of our results, to which end we provide all the code necessary.^23^ A clinical validation of the algorithm would also be desirable. However, establishing a gold standard in the clinical setting against which the algorithm can be compared is a major challenge. Some known sources of bias, as described previously, remain in our method, and we hope that further collaborative efforts towards refining the algorithm will be made.

## Conclusion

The algorithm presented in this study provides a novel solution to measuring ventilation volume, peak pressure, and ventilation rate during ongoing simulated CPR in an experimental setting. The algorithm is not reliant on any particular device, due to the use of raw waveform data. The algorithm can thus with adjustments be applied to other waveform data from ventilation during CPR, and could be further strengthened with external validation.

## CRediT authorship contribution statement

**Johan Mälberg:** Writing – review & editing, Writing – original draft, Visualization, Validation, Supervision, Resources, Project administration, Methodology, Investigation, Formal analysis, Data curation, Conceptualization. **Jeroen A. van Eijk:** Writing – review & editing, Visualization, Methodology, Investigation, Formal analysis. **Lotte C. Doeleman:** Writing – review & editing, Visualization, Methodology, Investigation, Formal analysis. **Patrick Schober:** Writing – review & editing, Visualization, Methodology, Investigation, Formal analysis. **Hans van Schuppen:** Writing – review & editing, Visualization, Methodology, Investigation, Formal analysis. **David Smekal:** Writing – review & editing, Writing – original draft, Validation, Supervision, Project administration, Methodology, Investigation, Formal analysis, Conceptualization. **Sten Rubertsson:** Writing – review & editing, Supervision, Resources, Project administration, Conceptualization. **Douglas Spangler:** Writing – review & editing, Writing – original draft, Visualization, Validation, Supervision, Software, Resources, Project administration, Methodology, Investigation, Formal analysis, Data curation, Conceptualization.

## Funding

This research did not receive any specific grant from funding agencies in the public, commercial, or not-for-profit sectors.

## Declaration of competing interest

The authors declare the following financial interests/personal relationships which may be considered as potential competing interests: Jeroen van Eijk reports a relationship with Stryker Emergency Care and Ambu A/S that includes: funding grants. Lotte Doeleman reports a relationship with Stryker Emergency Care that includes: funding grants. Hans van Schuppen reports a relationship with Stryker Emergency Care and Ambu A/S that includes: funding grants. Patrick Schober reports a relationship with Health Holland and Ambu A/S that includes: funding grants. If there are other authors, they declare that they have no known competing financial interests or personal relationships that could have appeared to influence the work reported in this paper.

## Appendix A Supplementary material

Supplementary file 1 Tests and corresponding ventilator settings used in the evaluation.

Supplementary file 2 Additional quantifications of performance of the algorithm and the standard method.
