# Supplementary material for: A novel algorithm to determine ventilation parameters during cardiopulmonary resuscitation using pneumotachography waveform data
Source: Resusc Plus. 2026 Jan 21;28:101238. doi: 10.1016/j.resplu.2026.101238 (PMC12886083; doi:10.1016/j.resplu.2026.101238)
Supplement: Supplementary file 2 — Additional quantifications of performance of the algorithm and the standard method. [file mmc2.zip › Supplemental file 2 revision.html]

Supplementary material 2 for: A method for determining ventilation parameters during ongoing CPR using pneumotachography waveform data


# Supplementary material 2 for: A method for determining ventilation parameters during ongoing CPR using pneumotachography waveform data

- Setup
- Load
  functions
- Set grid search parameters
- Load data
- Perform
  grid search
- Extract optimal parameters
- Extract optimal
  paramters using 2 given values
- Combine datasets for
  evaluation
- Generate
  figure 2
- Generate table
  2
- Generate
  figure 4
- Additional accuracy measures
  - Bland-Altman plots
  - Lin’s Concordance
    correlation
  - RMSE
  - Proportion
    with 10%
  - Generate waveforms for
    visual inspection
  - Generate sample
    waveforms from clinical data

# Setup

```
library(tidyverse)
```

```
## ── Attaching core tidyverse packages ──────────────────────── tidyverse 2.0.0 ──
## ✔ dplyr     1.1.4     ✔ readr     2.1.5
## ✔ forcats   1.0.0     ✔ stringr   1.5.1
## ✔ ggplot2   3.5.1     ✔ tibble    3.2.1
## ✔ lubridate 1.9.3     ✔ tidyr     1.3.1
## ✔ purrr     1.0.2     
## ── Conflicts ────────────────────────────────────────── tidyverse_conflicts() ──
## ✖ dplyr::filter() masks stats::filter()
## ✖ dplyr::lag()    masks stats::lag()
## ℹ Use the conflicted package (<http://conflicted.r-lib.org/>) to force all conflicts to become errors
```

```
library(knitr)
library(slider)
library(DescTools)
```

```
## Warning: package 'DescTools' was built under R version 4.4.3
```

```
library(lme4)
```

```
## Loading required package: Matrix
## 
## Attaching package: 'Matrix'
## 
## The following objects are masked from 'package:tidyr':
## 
##     expand, pack, unpack
```

```
options(scipen = 999)
knitr::opts_chunk$set(warning = FALSE,
                      message = FALSE)

sessionInfo()
```

```
## R version 4.4.2 (2024-10-31 ucrt)
## Platform: x86_64-w64-mingw32/x64
## Running under: Windows 11 x64 (build 22631)
## 
## Matrix products: default
## 
## 
## locale:
## [1] LC_COLLATE=English_Sweden.utf8  LC_CTYPE=English_Sweden.utf8   
## [3] LC_MONETARY=English_Sweden.utf8 LC_NUMERIC=C                   
## [5] LC_TIME=English_Sweden.utf8    
## 
## time zone: Europe/Stockholm
## tzcode source: internal
## 
## attached base packages:
## [1] stats     graphics  grDevices utils     datasets  methods   base     
## 
## other attached packages:
##  [1] lme4_1.1-35.5     Matrix_1.6-5      DescTools_0.99.60 slider_0.3.2     
##  [5] knitr_1.45        lubridate_1.9.3   forcats_1.0.0     stringr_1.5.1    
##  [9] dplyr_1.1.4       purrr_1.0.2       readr_2.1.5       tidyr_1.3.1      
## [13] tibble_3.2.1      ggplot2_3.5.1     tidyverse_2.0.0  
## 
## loaded via a namespace (and not attached):
##  [1] gld_2.6.8         gtable_0.3.4      xfun_0.41         bslib_0.6.1      
##  [5] lattice_0.21-9    tzdb_0.4.0        vctrs_0.6.5       tools_4.4.2      
##  [9] generics_0.1.3    proxy_0.4-27      fansi_1.0.6       pkgconfig_2.0.3  
## [13] data.table_1.15.0 readxl_1.4.3      lifecycle_1.0.4   rootSolve_1.8.2.4
## [17] compiler_4.4.2    Exact_3.3         munsell_0.5.0     htmltools_0.5.8.1
## [21] class_7.3-22      sass_0.4.9        yaml_2.3.8        nloptr_2.0.3     
## [25] pillar_1.9.0      jquerylib_0.1.4   MASS_7.3-60       cachem_1.0.8     
## [29] boot_1.3-28.1     nlme_3.1-163      tidyselect_1.2.1  digest_0.6.37    
## [33] mvtnorm_1.2-4     stringi_1.8.3     splines_4.4.2     fastmap_1.1.1    
## [37] grid_4.4.2        colorspace_2.1-0  lmom_3.2          expm_1.0-0       
## [41] cli_3.6.3         magrittr_2.0.3    utf8_1.2.4        e1071_1.7-14     
## [45] withr_3.0.0       scales_1.3.0      warp_0.2.1        timechange_0.3.0 
## [49] rmarkdown_2.25    httr_1.4.7        cellranger_1.1.0  hms_1.1.3        
## [53] evaluate_0.23     haven_2.5.4       rlang_1.1.4       Rcpp_1.0.12      
## [57] glue_1.8.0        minqa_1.2.6       rstudioapi_0.15.0 jsonlite_1.8.8   
## [61] R6_2.5.1          fs_1.6.5
```

# Load functions

```
# Function to check that all values within a given span are above a threshold
# Used to build the rectangle which check pressures for the start of a breath

check_lags <- function(data, n_lag, threshold){
  
  out <- rep(0,length(data))
  
  for(i in 1:length(data)){
    if(all(data[i:min(i+n_lag,
                      length(data))] > threshold)){
      out[i] <- sign(data[i])
    } 
  }
  
  return(out)
}

# get local mean value (for smoothing)

local_mean <- function(data, n_window){
  
  out <- rep(0,length(data))
  
  for(i in 1:length(data)){
    window <- data[max(i-n_window,1):
                   min(i+n_window,
                        length(data))]
    out[i] <- mean(window)
  } 
  
  return(out)
}

# get local derivative
# Looked at using the slope of the pressure to estimate variable lead times,
# Our efforts in this direction resulted in brittle lead time estimates that 
# were quite sensitive to artefacts, but the approach is appealing in principle!

local_slope <- function(data, n_window){
  
  out <- rep(0,length(data))
  
  for(i in 1:length(data)){
    window <- data[max(i-n_window,1):
                   min(i+n_window,
                        length(data))]
    
    out[i] <- first(window) - last(window)
  } 
  
  return(out)
}

# Function to parse raw data at the observation level, 256 samples per second

parse_data <- function(data,
                       params,
                       sample_rate = 256){
  out <- data  %>%
    group_by(file) %>%
    mutate(direction = check_lags(Paw,
                                   n_lag = params$paw_secs*sample_rate,
                                   threshold = params$paw_threshold),
           flow_abs_smooth = local_mean(abs(Flow),round(params$flow_smooth_secs*sample_rate/2)),
           CO2_delayed = lead(CO2,round(params$CO2_delay*sample_rate)),
           in_start = direction > 0 & (direction > lag(direction) | is.na(lag(direction))),
           in_start_corr = in_start & !slide_lgl(in_start,
                                                 ~ any(.x == T),
                                                 .before = round(params$gap_window_secs*sample_rate)+1,
                                                 .after = -1),
           breath_corr = cumsum(in_start_corr)
    ) %>%
    arrange(file,Time) %>%
    mutate(breath_corr = lead(breath_corr,round(sample_rate*params$breath_lead))) %>%
    group_by(file,breath_corr) %>%
    mutate(Volume_flow = cumsum(Flow)/sample_rate/60*1000,
           Volume_in = cumsum(pmax(Flow,0))/sample_rate/60*1000,
           Volume_ex = cumsum(pmin(Flow,0))/sample_rate/60*-1000,
           flow_abs_calib = flow_abs_smooth - min(head(flow_abs_smooth,
                                                       sample_rate*params$window_secs)),
           flow_abs_calib_delay = lag(flow_abs_calib, 
                                      round(params$inactive_delay*sample_rate)),
           breath_inactive = flow_abs_calib_delay < params$flow_abs_threshold &
             Paw < params$paw_threshold &  
             Time > min(Time) + params$flow_smooth_secs/2,
           breath_active = !cumsum(breath_inactive)>0
    ) %>%
    ungroup()
  
  return(out)
  
}

# Function to generate breath-level measures based on data passed from the parse_data function

generate_measures <- function(data,
                              params,
                              sample_rate = 256){
  
  out <- data %>%
    group_by(file,breath_corr) %>%
    summarise(
      Duration = max(Time) - min(Time),
      CO2 = max(CO2_delayed),
      CO2 = ifelse(CO2 < params$CO2_min,NA,CO2),
      Time = min(Time),  
      Volume_in = sum(Flow[Flow>0 & breath_active])/sample_rate/60*1000,
      Volume_ex = sum(Flow[Flow<0 & breath_active])/sample_rate/60*-1000,
      Flow_diff = Volume_in - Volume_ex,
      Flow = max(Flow[breath_active]),
      across(c(Volume_in,Volume_ex,Flow),~ifelse(Volume_in > params$Volume_max,NA,.x)),
      Paw_over_30 = sum(Paw >= 30)/sample_rate,
      Paw = max(Paw[breath_active])) %>%
    ungroup() %>%
    group_by(file) %>%
    filter(breath_corr > 2,
           breath_corr < max(breath_corr,na.rm=T)-2,
           !is.na(breath_corr)) %>%
    ungroup()
  
  return(out)
}
```

# Set grid search parameters

```
params_list <- list(paw_threshold = list(1,2,5,8,10), 
               paw_secs = c(0.1,0.2,0.3,0.4,0.5), 
               window_secs = 6,
               gap_window_secs = 0.3,
               breath_lead = list(0,0.1,0.2,0.3,0.4,0.5), 
               flow_smooth_secs = 1,
               flow_abs_threshold = list(-1,1,5,10), 
               CO2_delay = 0.5,
               CO2_min = 2,
               Volume_max = 1600,
               inactive_delay = list(0,0.1,0.2,0.3))

param_grid <- expand.grid(params_list)

param_grid <- as.data.frame(sapply(param_grid,unlist))
```

# Load data

```
test <- read_csv("test_parameters.csv") %>%
  transmute(file = file,
            fileid = str_split_i(file," ",1),
            mode = `Mode ventilator`,
            vent_type = vent_mode,
            Volume_in_true = Vti,
            Volume_ex_true = Vte,
            Paw_true = PIP,
            Frequency_true = VF,
            Duration_true = 60/VF)

files <- list.files("waveforms")

for(i in files){
  
  fn = paste0("waveforms/",i)
  
  file_data = read.delim(fn,skip = 5)[-1,] %>%
    filter(!is.na(Time)) %>%
    mutate(across(everything(),function(x) as.numeric(gsub(",",".",x))),
           file = i) %>%
    select(file,Time,Volume,Flow,Paw,CO2)
  
  if(i == first(files)){
    test_data_raw = file_data
  } else{
    test_data_raw = rbind(test_data_raw,file_data)
  }
  
}

flux_param_files <- list.files("flux_parameters")

for(i in flux_param_files){
  
  fn = paste0("flux_parameters/",i)
  
  flux_measure_data = read.delim(fn,skip = 4)[-1,] %>%
    select(Time = Tiempo,
           Volume_in = VTi,
           Volume_ex = VTe,
           Frequency = RR,
           Paw = PIP) %>%
    mutate(across(everything(),function(x) as.numeric(gsub(",",".",x))),
           Duration = lead(Time) - Time,
           file = i,
           fileid = str_split_i(i," ",1),
           breath_corr = row_number()) %>%
    filter(breath_corr>2,
           breath_corr< max(breath_corr-2)) %>%
    mutate(Volume_in = ifelse(Volume_in>params_list$Volume_max,NA,Volume_in),
           Volume_ex = ifelse(Volume_ex>params_list$Volume_max,NA,Volume_ex))
  
  if(i == first(flux_param_files)){
    flux_measures = flux_measure_data
  } else{
    flux_measures = rbind(flux_measures,flux_measure_data)
  }
  
}
```

# Perform grid search

```
# Set eval to TRUE ^ to process new data

for(i in 1:nrow(param_grid)){
  
  test_params <- as.list(unlist(param_grid[i,]))
  
  test_data <- parse_data(test_data_raw,test_params)
  
  test_measures <- generate_measures(test_data,test_params)
  
  test_measures <- bind_cols(data.frame(run = i),param_grid[i,],test_measures)
  
  if(i==1){
    out_measures <- test_measures
  } else{
    out_measures <- bind_rows(out_measures,test_measures)
  }
  print(i)
}

save(out_measures,file = "out_measures.rda")
```

Load data if already calculated

```
load("out_measures.rda")
```

# Extract optimal parameters

```
verify_measures <- out_measures %>%
  left_join(test,by = "file") %>%
  mutate(Volume_in_diff = Volume_in - Volume_in_true,
         Volume_ex_diff = Volume_ex - Volume_ex_true,
         Volume_in_dev = abs(Volume_in - Volume_in_true),
         Volume_ex_dev = abs(Volume_ex - Volume_ex_true),
         Paw_diff = Paw - Paw_true,
         Paw_dev = abs(Paw - Paw_true),
         Duration_diff = Duration - Duration_true,
         Duration_dev = abs(Duration - Duration_true),
         Volume_in_avg = (Volume_in+Volume_in_true)/2,
         Volume_ex_avg = (Volume_ex+Volume_ex_true)/2,
         Paw_avg = (Paw+Paw_true)/2,
         Duration_avg = (Duration+Duration_true)/2) 


grid_search_results <- verify_measures %>%
  group_by(run) %>%
  summarise(across(all_of(names(params_list)),first),
            vol_in_diff = mean(Volume_in_diff,na.rm = T),
            vol_ex_diff = mean(Volume_ex_diff,na.rm = T),
            vol_in_dev = mean(Volume_in_dev,na.rm = T),
            vol_ex_dev = mean(Volume_ex_dev,na.rm = T),
            paw_dev = mean(Paw_dev,na.rm = T),
            duration_dev = mean(Duration_dev,na.rm = T),
            vol_in_sd = sd(Volume_in,na.rm = T),
            vol_ex_sd = sd(Volume_ex,na.rm = T),
            paw_sd = sd(Paw,na.rm = T),
            duration_sd = sd(Duration,na.rm = T),
            vol_in_dev_sd = vol_in_dev / vol_in_sd,
            vol_ex_dev_sd = vol_ex_dev / vol_ex_sd,
            paw_dev_sd = paw_dev / paw_sd,
            duration_dev_sd = duration_dev / duration_sd,
            mean_dev = (vol_in_dev_sd+vol_ex_dev_sd+paw_dev_sd+duration_dev_sd)/4) %>%
  arrange(mean_dev)

params_optim <- as.list(grid_search_results[1,])
```

While functioning well in the ventilator data, the optimized
parameters were found to be too sensitive to handle artefacts in the
actual patient data. The parameters were thus tweaked based on the
patient data. They were then applied to test data to determine how well
the recovered the known ventilator settings.

# Extract optimal paramters using 2 given values

```
params_v2 <- grid_search_results %>%
  filter(paw_threshold == 8,
         flow_abs_threshold == 5) %>%
  filter(row_number() == 1) %>%
  as.list()
```

# Combine datasets for evaluation

```
test_data_v2 <- test_data_raw %>%
  parse_data(.,params_v2) %>%
  mutate(fileid = str_split_i(file," ",1))

test_measures_v2 <- test_data_v2 %>%
  generate_measures(.,params_v2) %>%
  mutate(fileid = str_split_i(file," ",1),
         Frequency = 60/Duration)

verify_measures_v2 <- test_measures_v2 %>%
  mutate(type = "Algorithm") %>%
  bind_rows(mutate(flux_measures,type ="Standard")) %>%
  left_join(test,by = "fileid") %>%
  mutate(Volume_in_diff = Volume_in - Volume_in_true,
         Volume_ex_diff = Volume_ex - Volume_ex_true,
         Volume_in_dev = abs(Volume_in - Volume_in_true),
         Volume_ex_dev = abs(Volume_ex - Volume_ex_true),
         Paw_diff = Paw - Paw_true,
         Paw_dev = abs(Paw - Paw_true),
         Duration_diff = Duration - Duration_true,
         Duration_dev = abs(Duration - Duration_true),
         Frequency_diff = Frequency - Frequency_true,
         Frequency_dev = abs(Frequency - Frequency_true),
         Volume_in_avg = (Volume_in+Volume_in_true)/2,
         Volume_ex_avg = (Volume_ex+Volume_ex_true)/2,
         Paw_avg = (Paw+Paw_true)/2,
         Duration_avg = (Duration+Duration_true)/2,
         Frequency_avg = (Frequency+Frequency_true)/2)
```

# Generate figure 2

```
fig2 <- test_data_v2 %>%
  ungroup() %>%
  select(file,
         Time,
         "Smoothed absolute Flow rate (calibrated)" = flow_abs_calib,
         "Flow rate" = Flow,
         "Pressure" = Paw,
         breath_active) %>%
  mutate(file = str_split_i(file," ",1)) %>%
  filter(Time > 17,
         Time < 23,
         file == 25
  ) %>%
  pivot_longer(-c(file,Time,breath_active)) %>% 
  ggplot(aes(x = Time,
             y = value,
             color = breath_active
  )) +
  geom_hline(yintercept = 0,
             color="darkgrey") +
  # geom_hline(yintercept = 5,
  #            color="darkgrey") +
  geom_point(size = 0.2) +
  facet_wrap(~name,
             scales="free",
             ncol = 1)  +
  ylab("L/min                                                   cmH2O                                                   L/min") +
  xlab("Seconds") +
  guides(color="none") +
  theme_minimal() +
  theme(strip.text = element_text(size = 11))

ggsave("outputs/fig2.tif",
       fig2,
       width = 6,
       height = 8)

fig2
```

# Generate figure 3

```
fig3 <- verify_measures_v2 %>%
  select(fileid,
         "Inspiratory volume" = Volume_in_diff,
         "Expiratory volume" = Volume_ex_diff,
         type,
         vent_type,
         "Peak Pressure" = Paw_diff,
         Frequency = Frequency_diff) %>%
  pivot_longer(-c(fileid,type,vent_type)) %>%
  mutate(name = factor(name,levels = unique(name))) %>%
  ggplot(aes(x=value,color=NULL,fill=type)) +
  scale_fill_manual(values = c("dodgerblue","orange")) + 
  geom_histogram(position = "dodge",bins = 20,linewidth = 0.25) +
  geom_vline(xintercept = 0) +
  facet_grid(vent_type~name,scales="free") +
  labs(x = "  ml                                    ml                                   cmH2O                         Per minute\nDifference between measured value and ventilator setting") +
  theme_minimal() +
  guides(fill="none") +
  theme(strip.text = element_text(size = 11))

ggsave("outputs/fig3.pdf",
       fig3,
       width = 8,
       height = 6)

fig3
```

# Generate table 2

```
est_ci_lme <- function(data,label){
  
   if(sum(!is.na(data[,label])) == 0){
     return(NA)
   }
     
  mod <- lmer(get(label) ~ 1 + (1|fileid),
                        data = data)
  
  ci <- confint(mod,method = "boot")
  
return(paste0(round(fixef(mod),1), " (",
      round(ci[3,1],1)," - ",
      round(ci[3,2],1),")"))
}

est_median_iqr <- function(data,label,r=0){
  
  perc <- quantile(data[,label],c(0.5,.25,0.75),na.rm=T)
  
  return(paste0(round(perc[1],r)," (",
               round(perc[2],r)," - ",
               round(perc[3],r),")"))
}

table2_base <- verify_measures_v2 %>%
  #filter(type == "Algorithm") %>%
  group_by(type,vent_type) %>%
  summarise(n_runs = length(unique(fileid)),
            n_breaths_volume_in = sum(!is.na(Volume_in_true)),
            n_breaths_volume_ex = sum(!is.na(Volume_ex_true)),
            n_breaths_pressure = sum(!is.na(Paw_true)),
            n_breaths_frequency = sum(!is.na(Frequency_true)),
            median_set_volume_in = median(Volume_in_true,na.rm=T),
            median_measured_volume_in = round(median(Volume_in[!is.na(Volume_in_true)],na.rm=T)),
            
            median_set_volume_ex = median(Volume_ex_true,na.rm=T),
            median_measured_volume_ex = round(median(Volume_ex[!is.na(Volume_ex_true)],na.rm=T)),
            
            median_set_pressure_in = median(Paw_true,na.rm=T),
            median_measured_pressure_in = median(Paw[!is.na(Paw_true)],na.rm=T),
            
            median_set_pressure = median(Paw_true,na.rm=T),
            median_measured_pressure = median(Paw[!is.na(Paw_true)],na.rm=T),
            
            median_set_Frequency = median(Frequency_true,na.rm=T),
            median_measured_Frequency = median(Frequency[!is.na(Frequency_true)],na.rm=T)) 

# Could also bootstrap some confidence intervals for the mean, but these models
# get very upset fitting these somewhat oddly distributed data with small sample sizes. 

# table2_mods <- verify_measures_v2 %>%
#   filter(type != "Standard") %>%
#   group_by(vent_type) %>%
#   do(volume_in_diff = est_ci_lme(.,label = "Volume_in_diff"),
#      Volume_ex_diff = est_ci_lme(.,label = "Volume_ex_diff"),
#      Paw_diff = est_ci_lme(.,label = "Paw_diff"),
#      Duration_diff = est_ci_lme(.,label = "Duration_diff"))

table2_iqrs <- verify_measures_v2 %>%
  #filter(type == "Algorithm") %>%
  group_by(type,vent_type) %>%
  do(volume_in_diff = est_median_iqr(.,label = "Volume_in_diff"),
     Volume_ex_diff = est_median_iqr(.,label = "Volume_ex_diff"),
     Paw_diff = est_median_iqr(.,label = "Paw_diff",r=1),
     Frequency_diff = est_median_iqr(.,label = "Frequency_diff",r=1)) %>%
  ungroup()

table2_pct_diffs <- verify_measures_v2 %>%
  #filter(type == "Algorithm") %>%
  group_by(type,vent_type) %>%
  summarise(Volume_in_pct_diff = median(Volume_in_diff/Volume_in_avg,na.rm=T)*100,
         Volume_ex_pct_diff = median(Volume_ex_diff/Volume_ex_avg,na.rm=T)*100,
         Paw_pct_diff = median(Paw_diff/Paw_avg,na.rm=T)*100,
         Frequency_pct_diff = median(Frequency_diff/Frequency_avg,na.rm=T)*100) %>%
  ungroup()

  

table2 <- table2_base %>%
  bind_cols(select(table2_iqrs,-vent_type,-type)) %>%
  bind_cols(select(table2_pct_diffs,-vent_type,-type)) %>%
  ungroup()


# Did some hand formatting of the tables in excel after this.

kable(t(table2))
```

|  |  |  |  |  |  |  |
| --- | --- | --- | --- | --- | --- | --- |
| type | Algorithm | Algorithm | Algorithm | Standard | Standard | Standard |
| vent\_type | Asynchronous | No compressions | Synchronous (30:2) | Asynchronous | No compressions | Synchronous (30:2) |
| n\_runs | 10 | 20 | 7 | 10 | 20 | 7 |
| n\_breaths\_volume\_in | 189 | 637 | 51 | 501 | 635 | 149 |
| n\_breaths\_volume\_ex | 92 | 637 | 51 | 173 | 635 | 149 |
| n\_breaths\_pressure | 189 | 637 | 51 | 501 | 635 | 149 |
| n\_breaths\_frequency | 189 | 637 | 0 | 501 | 635 | 0 |
| median\_set\_volume\_in | 400 | 500 | 500 | 334 | 500 | 510 |
| median\_measured\_volume\_in | 396 | 445 | 423 | 63 | 444 | 26 |
| median\_set\_volume\_ex | 413 | 399 | 299 | 245 | 399 | 526 |
| median\_measured\_volume\_ex | 399 | 388 | 348 | 330 | 383 | 22 |
| median\_set\_pressure\_in | 36 | 23 | 22 | 32 | 23 | 28 |
| median\_measured\_pressure\_in | 37.1 | 22 | 21.4 | 19.8 | 22 | 12.3 |
| median\_set\_pressure | 36 | 23 | 22 | 32 | 23 | 28 |
| median\_measured\_pressure | 37.1 | 22 | 21.4 | 19.8 | 22 | 12.3 |
| median\_set\_Frequency | 10 | 20 | NA | 10 | 20 | NA |
| median\_measured\_Frequency | 10.0065147414224 | 20.026075619296 | NA | 54.9 | 20 | NA |
| volume\_in\_diff | 5 (-28 - 32) | -10 (-55 - 22) | -46 (-76 - 10) | -189 (-294 - -44) | -18 (-63 - 20) | -484 (-487 - -77) |
| Volume\_ex\_diff | -8 (-43 - 68) | -22 (-44 - 24) | -5 (-30 - 28) | -52 (-196 - 17) | -31 (-58 - 10) | -504 (-507 - -42) |
| Paw\_diff | 3 (0.3 - 5.8) | 0.1 (-1.4 - 0.5) | -1 (-1.9 - -0.2) | -2.9 (-32 - 1.8) | 0.1 (-1.5 - 0.5) | -15.6 (-17.1 - -1.9) |
| Frequency\_diff | 0 (0 - 0.1) | 0 (0 - 0.1) | NA (NA - NA) | 44.9 (20.3 - 73.2) | 0 (0 - 0) | NA (NA - NA) |
| Volume\_in\_pct\_diff | 1.1425753154412 | -3.25257043045612 | -13.5320367564261 | -121.621621621622 | -5.02311895754519 | -180.597014925373 |
| Volume\_ex\_pct\_diff | -1.64726461658043 | -5.84182947093748 | -0.763687469536664 | -13.2796780684105 | -9.37369257802694 | -183.941605839416 |
| Paw\_pct\_diff | 10.3448275862069 | 0.49875311720699 | -4.91803278688524 | -13.0750605326876 | 0.369685767097972 | -77.2277227722772 |
| Frequency\_pct\_diff | 0.0651253663301768 | 0.0977039570102647 | NA | 138.366718027735 | 0 | NA |

# Generate figure 4

```
viz_time_min = 30
viz_time_max = 70
#filenr = sample(str_split_i(test$file," ",1),2)
filenr = c(33,12)

fig4 <- test_data_v2 %>%
  ungroup() %>%
  select(file,
         Time,
         "Volume (Inspiratory)" = Volume_in,
         "Volume (Expiratory)" = Volume_ex,
         Flow,
         "Pressure" = Paw,
         breath_active) %>%
  mutate(file = str_split_i(file," ",1)) %>%
  filter(Time > min(Time) + viz_time_min,
         Time < min(Time) + viz_time_max,
         file %in% filenr
  ) %>%
  pivot_longer(-c(file,Time,breath_active)) %>% 
  ggplot(aes(x = Time,
             y = value,
             color = breath_active
  )) +
  geom_hline(yintercept = 0,
             color="darkgrey") +
  geom_point(size = 0.1) +
  #scale_color_manual(values = c("orange","darkgreen")) +
  facet_grid(name~file,
             scales = "free_y") +
  geom_point(data = pivot_longer(filter(transmute(test_measures_v2,
                                                  file = str_split_i(file," ",1),
                                                  Time,
                                                  "Volume (Inspiratory)" = Volume_in,
                                                  "Volume (Expiratory)" = Volume_ex,
                                                  Pressure = Paw,
                                                  Frequency),
                                        Time > min(Time) + viz_time_min,
                                        Time < min(Time) + viz_time_max,
                                        file %in% filenr
  ),
  -c(file,Time)),
  shape = 5,
  color = "black"
  ) +
  geom_hline(data = filter(pivot_longer(transmute(test,
                                                  file = str_split_i(file," ",1),
                                                  "Volume (Inspiratory)" = Volume_in_true,
                                                  "Volume (Expiratory)" = Volume_ex_true,
                                                  Pressure = Paw_true,
                                                  Frequency = Frequency_true),
                                        -file),
                           file %in% filenr
  ),
  aes(yintercept = value),
  color = "black"
  )+
  facet_grid(name~file,
             scales = "free_y") + 
  scale_x_continuous(breaks=seq(0, max(test_data_v2$Time), 10)) +
  theme_minimal() +
  theme(panel.grid.major.y = element_blank(),
        panel.grid.minor.y = element_blank()) +
  guides(color="none") +
  ylab("ml                            ml                           cmH2O                          Per minute                    L/min") +
  xlab("Seconds") +
  theme(strip.text = element_text(size = 11))

ggsave("outputs/fig4.tif",
       fig4,
       width = 8,
       height = 8)

fig4
```

# Additional accuracy measures

## Bland-Altman plots

```
diffs <- verify_measures_v2 %>%
  select(fileid,
         type,
         vent_type,
         Volume_in_diff,
         Volume_ex_diff,
         Paw_diff,
         Frequency_diff) %>%
  pivot_longer(ends_with("diff"),
               values_to = "diff") %>%
  mutate(name = gsub("_diff","",name))

ba <- verify_measures_v2 %>%
  select(fileid,
         type,
         vent_type,
         Volume_in_avg,
         Volume_ex_avg,
         Paw_avg,
         Frequency_avg) %>%
  pivot_longer(ends_with("_avg"),
               values_to = "avg") %>%
  mutate(name = gsub("_avg","",name)) %>%
  bind_cols(select(diffs,diff)) %>%
  group_by(type,vent_type,name) %>%
  mutate(lo = sd(diff,na.rm=T)*-1.96,
         hi = sd(diff,na.rm=T)*1.96,
         name = case_when(name == "Volume_in" ~ "Inspiratory volume",
                          name == "Volume_ex" ~ "Expiratory volume",
                          name == "Paw" ~ "Peak Pressure",
                          .default = name)) %>%
  ungroup() %>%
  filter(!is.na(avg))


ba %>%
  ggplot(aes(x=avg,y=diff,color = type)) +
  scale_color_manual(values = c("dodgerblue","orange")) + 
  geom_point() +
  geom_hline(aes(yintercept = lo)) +
  geom_hline(aes(yintercept = hi)) +
  facet_grid(vent_type~name+type,scales="free") +
  theme_minimal() +
  labs(x= "Average value",
       y = "Difference")
```

```
ba %>%
  group_by(type,vent_type,name) %>%
  summarise(across(c(avg,diff,lo,hi),mean)) %>% 
  kable()
```

| type | vent\_type | name | avg | diff | lo | hi |
| --- | --- | --- | --- | --- | --- | --- |
| Algorithm | Asynchronous | Expiratory volume | 390.40148 | -2.0883435 | -168.1697923 | 168.1697923 |
| Algorithm | Asynchronous | Frequency | 10.70243 | 0.0291903 | -0.5086964 | 0.5086964 |
| Algorithm | Asynchronous | Inspiratory volume | 391.89724 | 16.4928833 | -131.9043761 | 131.9043761 |
| Algorithm | Asynchronous | Peak Pressure | 34.34577 | 2.6915344 | -9.3160519 | 9.3160519 |
| Algorithm | No compressions | Expiratory volume | 383.00039 | -14.0651634 | -79.4238508 | 79.4238508 |
| Algorithm | No compressions | Frequency | 21.64779 | 0.0302799 | -0.0936708 | 0.0936708 |
| Algorithm | No compressions | Inspiratory volume | 439.36903 | -18.5146847 | -90.3146811 | 90.3146811 |
| Algorithm | No compressions | Peak Pressure | 24.65463 | 0.1318681 | -4.2587511 | 4.2587511 |
| Algorithm | Synchronous (30:2) | Expiratory volume | 367.74611 | -8.5470027 | -135.1474237 | 135.1474237 |
| Algorithm | Synchronous (30:2) | Inspiratory volume | 405.96227 | -34.3499796 | -104.1245745 | 104.1245745 |
| Algorithm | Synchronous (30:2) | Peak Pressure | 21.47255 | -1.9568627 | -5.4447452 | 5.4447452 |
| Standard | Asynchronous | Expiratory volume | 322.41776 | -62.0328947 | -372.9138922 | 372.9138922 |
| Standard | Asynchronous | Frequency | 33.95679 | 47.3147705 | -62.1609240 | 62.1609240 |
| Standard | Asynchronous | Inspiratory volume | 284.14739 | -164.2040816 | -507.6833193 | 507.6833193 |
| Standard | Asynchronous | Peak Pressure | 25.44591 | -14.2858283 | -35.4357950 | 35.4357950 |
| Standard | No compressions | Expiratory volume | 383.40032 | -15.6962025 | -116.0165161 | 116.0165161 |
| Standard | No compressions | Frequency | 21.63575 | -0.0670866 | -0.7663424 | 0.7663424 |
| Standard | No compressions | Inspiratory volume | 438.74209 | -23.2436709 | -94.3458653 | 94.3458653 |
| Standard | No compressions | Peak Pressure | 24.55197 | -0.0866142 | -5.5723435 | 5.5723435 |
| Standard | Synchronous (30:2) | Expiratory volume | 309.33103 | -324.9931034 | -477.2098848 | 477.2098848 |
| Standard | Synchronous (30:2) | Inspiratory volume | 318.85862 | -321.3172414 | -432.2247942 | 432.2247942 |
| Standard | Synchronous (30:2) | Peak Pressure | 20.21946 | -11.5744966 | -15.3356380 | 15.3356380 |

#Boxplot of comparison of errors between Algorithm and Standard
method

```
verify_measures_v2 %>%
  select(fileid,
         "Inspiratory volume" = Volume_in_diff,
         "Expiratory volume" = Volume_ex_diff,
         type,
         vent_type,
         "Peak Pressure" = Paw_diff,
         Frequency = Frequency_diff) %>%
  pivot_longer(-c(fileid,type,vent_type)) %>%
  mutate(name = factor(name,levels = unique(name))) %>%
  ggplot(aes(x=value,color=NULL,fill=type)) +
  scale_fill_manual(values = c("dodgerblue","orange")) + 
  geom_boxplot() +
  geom_vline(xintercept = 0) +
  facet_grid(vent_type~name,scales="free") +
  labs(x = "  ml                                    ml                                   cmH2O                         Per minute\nDifference between measured value and ventilator setting") +
  theme_minimal() +
  guides(fill="none") +
  theme(strip.text = element_text(size = 11))
```

## Lin’s Concordance correlation

```
verify_measures_v2 %>%
  #filter(type == "Algorithm") %>%
  group_by(type,vent_type) %>%
  do(volume_in = CCC(.$Volume_in,.$Volume_in_true,na.rm=T)$rho.c[[1]],
     volume_ex = CCC(.$Volume_ex,.$Volume_ex_true,na.rm=T)$rho.c[[1]],
     Paw = CCC(.$Paw,.$Paw_true,na.rm=T)$rho.c[[1]],
     Frequency = CCC(.$Frequency,.$Frequency_true,na.rm=T)$rho.c[[1]]) %>%
  ungroup() %>%
  kable()
```

| type | vent\_type | volume\_in | volume\_ex | Paw | Frequency |
| --- | --- | --- | --- | --- | --- |
| Algorithm | Asynchronous | 0.766984 | 0.6882719 | 0.7428654 | 0.9887448 |
| Algorithm | No compressions | 0.9159057 | 0.9530749 | 0.9596644 | 0.9999743 |
| Algorithm | Synchronous (30:2) | 0.8466609 | 0.9024618 | 0.8216492 | NA |
| Standard | Asynchronous | 0.149248 | 0.4981046 | 0.05709396 | -0.007165341 |
| Standard | No compressions | 0.9023855 | 0.9158626 | 0.9317021 | 0.9987282 |
| Standard | Synchronous (30:2) | -0.04585426 | -0.05968676 | -0.01522615 | NA |

## RMSE

```
verify_measures_v2 %>%
  #filter(type == "Algorithm") %>%
  group_by(type,vent_type) %>%
  do(volume_in = RMSE(.$Volume_in,.$Volume_in_true,na.rm=T),
     volume_ex = RMSE(.$Volume_ex,.$Volume_ex_true,na.rm=T),
     Paw = RMSE(.$Paw,.$Paw_true,na.rm=T),
     Frequency = RMSE(.$Frequency,.$Frequency_true,na.rm=T)) %>%
  ungroup() %>%
  kable()
```

| type | vent\_type | volume\_in | volume\_ex | Paw | Frequency |
| --- | --- | --- | --- | --- | --- |
| Algorithm | Asynchronous | 69.11652 | 85.35888 | 5.4513 | 0.2604921 |
| Algorithm | No compressions | 49.62587 | 42.8639 | 2.175127 | 0.05654455 |
| Algorithm | Synchronous (30:2) | 62.82377 | 68.80632 | 3.375634 | NaN |
| Standard | Asynchronous | 306.4364 | 199.5235 | 23.02825 | 56.943 |
| Standard | No compressions | 53.41949 | 61.19258 | 2.842113 | 0.3964011 |
| Standard | Synchronous (30:2) | 389.281 | 405.5755 | 13.95628 | NaN |

## Proportion with 10%

```
pct_within_pct <- function(data,reference,pct = 0.1){
  ref <- mean(reference,na.rm=T)*pct
  mean(abs(data[!is.na(reference)])<ref,na.rm=T)
}

verify_measures_v2 %>%
  #filter(type == "Algorithm") %>%
  group_by(type,vent_type) %>%
  do(volume_in = pct_within_pct(.$Volume_in_diff,.$Volume_in_true),
     volume_ex = pct_within_pct(.$Volume_ex_diff,.$Volume_ex_true),
     Paw = pct_within_pct(.$Paw_diff,.$Paw_true),
     Frequency = pct_within_pct(.$Frequency_diff,.$Frequency_true)) %>%
  ungroup() %>%
  kable()
```

| type | vent\_type | volume\_in | volume\_ex | Paw | Frequency |
| --- | --- | --- | --- | --- | --- |
| Algorithm | Asynchronous | 0.6243386 | 0.3586957 | 0.4285714 | 0.994709 |
| Algorithm | No compressions | 0.6766091 | 0.6405024 | 0.9686028 | 1 |
| Algorithm | Synchronous (30:2) | 0.3529412 | 0.5490196 | 0.8235294 | NaN |
| Standard | Asynchronous | 0.170068 | 0.2368421 | 0.3453094 | 0.08183633 |
| Standard | No compressions | 0.6471519 | 0.4794304 | 0.9700787 | 0.992126 |
| Standard | Synchronous (30:2) | 0.1862069 | 0.1862069 | 0.3020134 | NaN |

## Generate waveforms for visual inspection

```
  d_long <- test_data_v2 %>%
  ungroup() %>%
  select(file,
         Time,
         "Volume (Inspiratory)" = Volume_in,
         "Volume (Expiratory)" = Volume_ex,
         Flow,
         "Pressure" = Paw,
         breath_active) %>%
  mutate(file = str_split_i(file," ",1)) %>%
  pivot_longer(-c(file,Time,breath_active))
  
  m_long <- pivot_longer(transmute(test_measures_v2,
                                    file = str_split_i(file," ",1),
                                    Time,
                                    "Volume (Inspiratory)" = Volume_in,
                                    "Volume (Expiratory)" = Volume_ex,
                                    Pressure = Paw,
                                    Frequency), -c(file,Time))
  
  t_long <- pivot_longer(transmute(test,
                                  file = str_split_i(file," ",1),
                                  "Volume (Inspiratory)" = Volume_in_true,
                                  "Volume (Expiratory)" = Volume_ex_true,
                                  Pressure = Paw_true,
                                  Frequency = Frequency_true),
                                -file)

for(i in 1:max(as.numeric(test_data_v2$fileid))){
  
  filenr = i
  


  samp <- filter(d_long, file %in% filenr) %>% 
  ggplot(aes(x = Time,
             y = value,
             color = breath_active
  )) +
  geom_hline(yintercept = 0,
             color="darkgrey") +
  geom_point(size = 0.1) +
  facet_grid(name~file,
             scales = "free_y") +
  geom_point(data = filter(m_long, file %in% filenr),
  shape = 5,
  color = "black"
  ) +
  geom_hline(data = filter(t_long, file %in% filenr),
  aes(yintercept = value),
  color = "black"
  )+
  facet_grid(name~file,
             scales = "free_y") + 
  #scale_x_continuous(breaks=seq(0, max(test_data_v2$Time), 10)) +
  theme_minimal() +
  theme(panel.grid.major.y = element_blank(),
        panel.grid.minor.y = element_blank())
  
  ggsave(paste0("outputs/waveform_images/",filenr,".tif"),
       samp,
       width = max(filter(d_long, 
                          file %in% filenr)$Time)/15,
       height = 8)
  
  
}
```

## Generate sample waveforms from clinical data

8 randomly selected observations from the clinical dataset are
provided below to demonstrate the issue identified upon applying the
optimized paramters to the clinical data. Observe the false positive
ventilations identified using the optimized parameters particularly in
the 2nd, 3rd and 4th samples, characterized by unreasonably low
pressures, small valumes and high ventilation frequencies. This issue is
substantially improved using the Adjusted parameters.

```
study_dir = "C:/Arbetsmaterial_Douglas/Analysforfragningar/fluxmed/data/Run251203/"

crf <- readxl::read_xlsx("C:/Arbetsmaterial_Douglas/Analysforfragningar/fluxmed/data/Master ventcrf 251203 edit.xlsx") %>%
  mutate(file = paste0(file_name," Signals.txt"))

study_files <- list.files(study_dir)

study_files <- study_files[study_files %in% filter(crf, is.na(exclude_variable))$file]

set.seed(42)

load_files = sample(study_files,8)

for(i in load_files){
  
  fn = paste0(study_dir,i)
  
  crf_i <- filter(crf,file == i) %>%
    filter(row_number()==1)
  
  study_file_data = read.delim(fn,skip = 5)[-1,] %>%
    mutate(across(everything(),function(x) as.numeric(gsub(",",".",x))),
           file = i) %>%
    filter(!is.na(Time),
           Time > crf_i$inclusion_start_time & 
           Time < crf_i$inclusion_stop_time) %>%
    select(file,Time,Volume,Flow,Paw,CO2)
  

  
  if(i == first(load_files)){
    study_data_raw = study_file_data
  } else{
    study_data_raw = rbind(study_data_raw,study_file_data)
  }
  
}

study_data_optim <- study_data_raw %>%
  parse_data(.,params_optim) %>%
  mutate(fileid = str_split_i(file," ",1))

study_measures_optim <- study_data_optim %>%
  generate_measures(.,params_optim) %>%
  mutate(fileid = str_split_i(file," ",1),
         Frequency = 60/Duration)

study_data_v2 <- study_data_raw %>%
  parse_data(.,params_v2) %>%
  mutate(fileid = str_split_i(file," ",1))

study_measures_v2 <- study_data_v2 %>%
  generate_measures(.,params_v2) %>%
  mutate(fileid = str_split_i(file," ",1),
         Frequency = 60/Duration)

study_data_long <- bind_rows(bind_cols(study_data_optim,params="Optimized"),
                        bind_cols(study_data_v2,params="Adjusted")) %>%
  ungroup() %>%
  select(file,
         Time,
         "Volume (Inspiratory)" = Volume_in,
         "Volume (Expiratory)" = Volume_ex,
         Flow,
         "Pressure" = Paw,
         breath_active,
         params) %>%
  mutate(file = str_split_i(file," ",1)) %>%
  pivot_longer(-c(file,Time,breath_active,params))


study_measures_long <- bind_rows(bind_cols(study_measures_optim,params="Optimized"),
                        bind_cols(study_measures_v2,params="Adjusted")) %>%
  transmute(file = str_split_i(file," ",1),
            Time,
            "Volume (Inspiratory)" = Volume_in,
            "Volume (Expiratory)" = Volume_ex,
            Pressure = Paw,
            Frequency,
            params) %>%
  pivot_longer(-c(file,Time,params))


for(i in unique(study_measures_long$file)){
  
  print(filter(study_data_long,
               file == i,
               abs(value)<1000) %>% 
  ggplot(aes(x = Time,
             y = value,
             color = breath_active
  )) +
  geom_hline(yintercept = 0,
             color="darkgrey") +
  geom_point(size = 0.1) +
  facet_grid(name~params,
             scales = "free_y") +
  geom_point(data = filter(study_measures_long,
                           file == i,
               abs(value)<1000),
             shape = 5,
             color = "black") +
  #scale_x_continuous(breaks=seq(0, max(test_data_v2$Time), 10)) +
  theme_minimal() +
  theme(panel.grid.major.y = element_blank(),
        panel.grid.minor.y = element_blank()))

  }
```
